# Exquisite Radiosensitivity Leading to a Diagnosis of Ph+MPAL Mimicking Cerebrospinal Fluid Dissemination of Intraventricular Meningioma Following Stereotactic Radiotherapy

**DOI:** 10.7759/cureus.107253

**Published:** 2026-04-17

**Authors:** Shinichiro Mizumatsu, Naoto Imoto, Souichi Akamine, Hisato Hatakeyama, Yasuhiro Hayashi

**Affiliations:** 1 Department of Radiology, Narita Memorial Hospital, Toyohashi, JPN; 2 Department of Neurosurgery, Narita Memorial Hospital, Toyohashi, JPN; 3 Cerebrospinal Center, Aoyama General Hospital, Toyokawa, JPN; 4 Department of Hematology and Oncology, Toyohashi Municipal Hospital, Toyohashi, JPN

**Keywords:** cerebrospinal fluid (csf) dissemination, dasatinib, diagnostic clue, elderly patient, exquisite radiosensitivity, intraventricular meningioma (ivm), mixed phenotype acute leukemia (mpal), philadelphia chromosome-positive (ph+), stereotactic radiotherapy (srt), therapy-related leukemia (trl)

## Abstract

We report a rare case of Philadelphia chromosome-positive (Ph+) mixed phenotype acute leukemia (MPAL) that initially radiologically mimicked cerebrospinal fluid (CSF) dissemination after resection and subsequent stereotactic radiotherapy (SRT) for an intraventricular meningioma. A 77-year-old woman underwent resection of an asymptomatic World Health Organization grade I lateral ventricular meningioma. Due to residual tumor growth, the patient received three sessions of SRT for seven lesions between 28 and 45 months postoperatively. Two of these lesions were radiologically diagnosed as CSF dissemination of meningioma. At 63 months postoperatively, 2 new frontal lobe lesions appeared and were similarly treated with SRT (21 Gy in 3 fractions). Follow-up magnetic resonance imaging revealed complete resolution of these new lesions only two months after this low-dose SRT. This remarkably rapid response was inconsistent with the typical radiobiology of meningioma, prompting diagnostic reconsideration. The patient was subsequently diagnosed with Ph+MPAL. Although chemotherapy (prednisolone and dasatinib) was discontinued after one month due to adverse effects, the patient achieved complete remission. No recurrence of Ph+MPAL was observed for 23 months after the discontinuation of chemotherapy, until the patient died of causes unrelated to the primary disease. The unexpectedly exquisite radiosensitivity should prompt clinicians to reconsider the initial diagnosis. Such rapid responses may indicate underlying hematologic malignancies rather than slow-growing tumors like meningioma. Even in elderly patients, early detection can lead to successful remission with short-term treatment.

## Introduction

Intraventricular meningiomas (IVMs) are rare, accounting for 0.5%-2% of all intracranial meningiomas, with approximately 80% arising in the trigone of the lateral ventricle, more commonly on the left side [1]. Although World Health Organization (WHO) grade I meningiomas are benign, rare cases of cerebrospinal fluid (CSF) dissemination have been reported, particularly following surgical resection, radiotherapy, or chemotherapy. Stereotactic radiotherapy (SRT) is widely used for the management of brain tumors [2]. However, since meningiomas typically exhibit low radiosensitivity, a rapid radiological response to SRT is unexpected and should prompt clinicians to reconsider the diagnosis.

Mixed phenotype acute leukemia (MPAL) is a rare form of acute leukemia characterized by differentiation into two or more hematopoietic cell lineages [3]. In the fifth edition of the WHO Classification of Hematopoietic Tumors, MPAL and acute leukemia of ambiguous lineage are classified as a single category based on shared clinical features, immunophenotype, and pathogenic mechanisms, with MPAL defined by blasts expressing markers of more than one hematopoietic lineage [4]. Philadelphia chromosome-positive (Ph+) MPAL is an especially rare subtype, representing less than 1% of adult acute leukemia cases, and is generally associated with poor prognosis. Furthermore, the incidence of therapy-related leukemia (TRL) is rising as the number of long-term cancer survivors increases, often resulting from DNA damage induced by prior radiation or cytotoxic drugs, a consideration relevant to this case, given the patient's multiple SRT sessions. Here, we report a rare case of Ph+MPAL that radiologically mimicked CSF dissemination of an IVM.

In this case, a disproportionately rapid and complete resolution after SRT served as the "diagnostic clue" that led to the suspicion of a hematologic malignancy rather than a slow-growing meningioma.

## Case presentation

**Figure 1:**
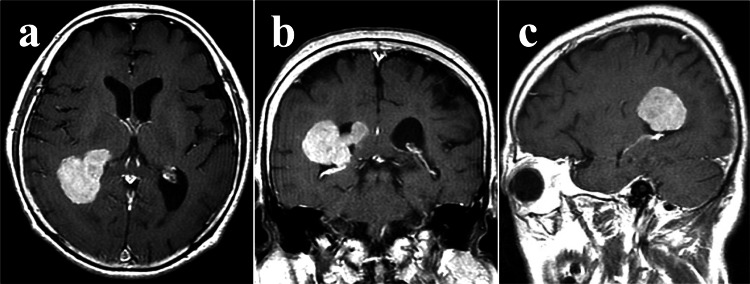
Preoperative gadolinium-enhanced MRI showing a meningioma in the right posterior horn of the lateral ventricle. (a) Axial, (b) Coronal, and (c) Sagittal views MRI; magnetic resonance imaging

A 77-year-old woman was incidentally diagnosed with a right lateral ventricle tumor and underwent gross total resection (Simpson grade I) (Figures 1a-1c).

**Figure 2:**
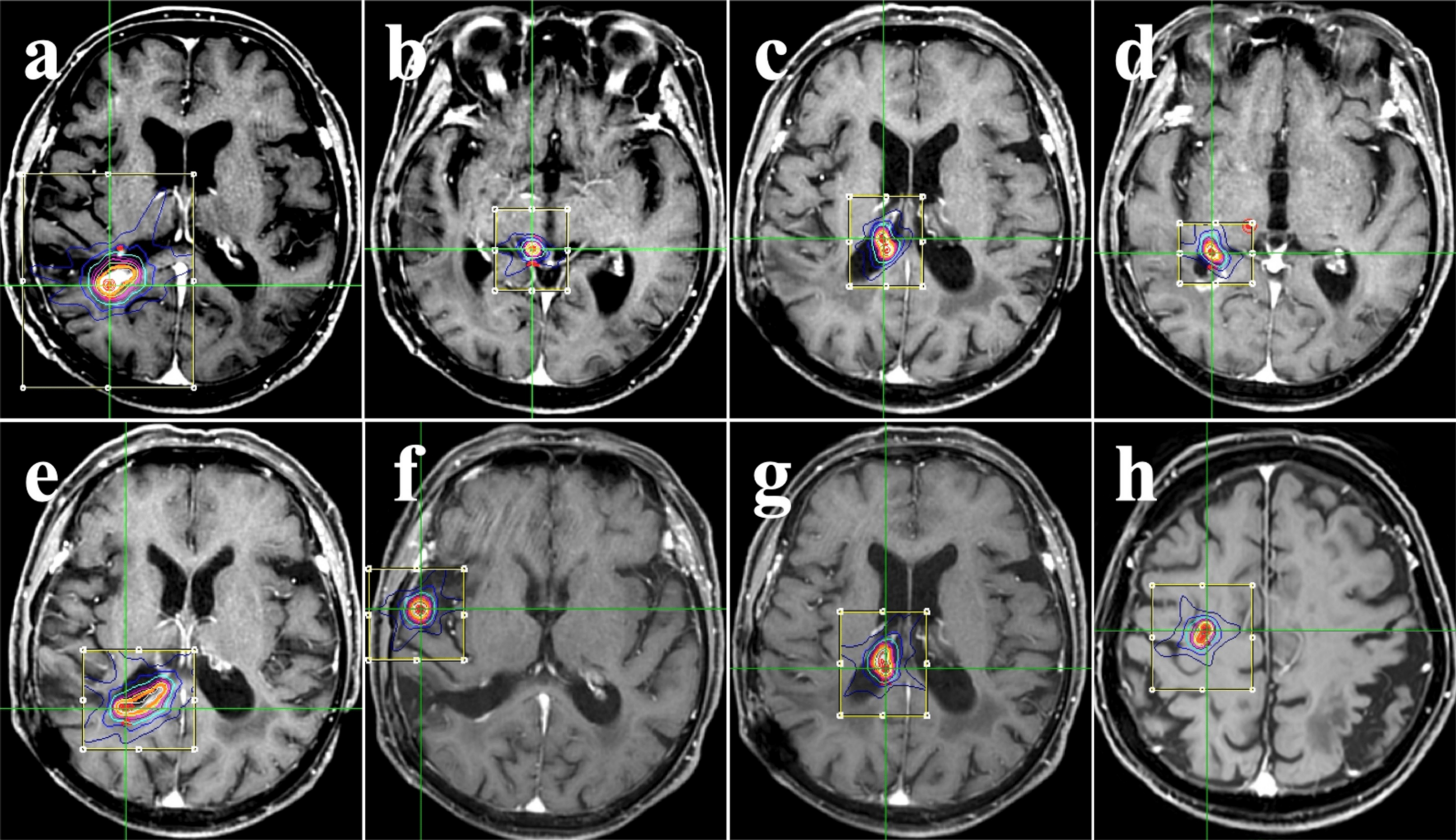
Treatment planning for SRT using CyberKnife®. Representative dose distributions generated by MultiPlan® (Accuray Incorporated, Sunnyvale, USA). Panels (a) through (h) correspond to the treatment sites listed in Table 1. (a) First SRT session: target lesion within the resection cavity. (b-e) Second SRT session: (b) a lesion distant from the resection cavity and (c–e) three lesions continuous with the cavity. (f, g) Third SRT session: (f) a lesion distant from the cavity and (g) a lesion continuous with the cavity (same as in panel c). (h) Fourth SRT session:  a single target volume covering two new frontal lobe lesions. SRT; stereotactic radiotherapy

Histopathology confirmed a transitional meningioma (WHO grade I). At 19 months postoperatively, a local recurrence developed within the resection cavity. At 28 months postoperatively, although the patient remained asymptomatic, the patient underwent SRT using CyberKnife (CK) (Accuray Inc., Sunnyvale, CA, USA) for the lesion in the posterior horn of the right lateral ventricle (Figure 2a) (Table 1).

**Table 1:** Summary of treatment parameters and dosimetric data for each SRT session (b) Every other day: SRT because of risk reduction for the midbrain SRT; stereotactic radiotherapy, D95; the dose covering 95% of the target volume

Date of SRT	Figure 2	Location	Initial/Salvage SRT	Target volume (ml)	Prescription dose (Gy)	Fractions	Maximum dose (Gy)	D95 isodose (%)	Treatment duration (days)
2020 March	(a)	Right lateral ventricular posterior horn	Initial	1.35	21	3	28.8	73	3
2020 October	(b)	Right midbrain	Initial	0.10	21	3	31.3	67	5
	(c)	Right lateral ventricular body	Initial	0.34	21	3	35.6	59	3
	(d)	Right lateral ventricular posterior horn	Initial	0.32	21	3	33.3	63	3
	(e)	Right lateral ventricular body	Initial	0.63	21	3	30.0	70	3
2021 August	(f)	Right temporal lobe	Initial	0.31	30	3	48.4	62	3
	(g)	Right lateral ventricular body	Salvage	0.45	27	3	44.3	61	3
2023 February	(h)	Right frontal lobe	Initial	0.08	21	3	26.6	79	3

**Figure 3:**
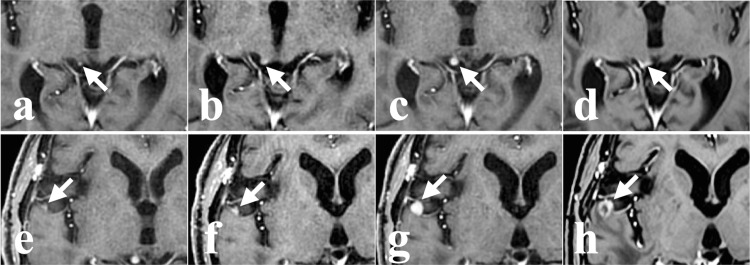
Radiographic evolution of presumed CSF dissemination on axial enhanced MRI (a-d) Right midbrain lesion and (e-h) right temporal lobe lesion at various time points post-surgery. White arrows indicate the tumors. (a) At 28 months, a small new lesion was retrospectively identified. (b) At 35 months, before SRT for the enlarging lesion. (c) At 37 months, the lesion showed a roundish expansion. (d) At 63 months, the lesion had collapsed. (e) At 37 months, a small new lesion was retrospectively identified. (f) At 40 months, showing lesion growth. (g) At 45 months, before SRT. (h) At 65 months, MRI showing central necrosis within the lesion. CSF; cerebrospinal fluid, MRI; magnetic resonance imaging, SRT; stereotactic radiotherapy

At 35 months postoperatively, further recurrences prompted a second SRT on four lesions (Figures 2b-2e). Three lesions were within the resection cavity (Figures 2c-2e), and one was located on the right midbrain surface, distant from the cavity (Figure 2b). At 45 months postoperatively, a third SRT was performed on two lesions (Figures 2f, 2g). One lesion was located on the temporal lobe surface of the right Sylvian fissure and was distant from the resection cavity (Figure 2f). The other was re-irradiated because of poor therapeutic effect after the second SRT (Figure 2g). The two superficial lesions distant from the resection cavity were slowly growing, elevated lesions, and were diagnosed radiologically as CSF dissemination from meningioma, though no biopsy was performed to confirm this diagnosis (Figures 3a, 3b, 3e-3g).

**Figure 4:**
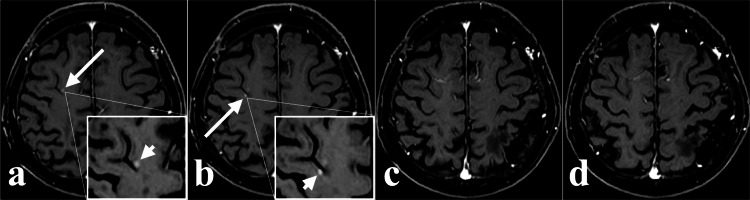
Rapid response of the right frontal lobe lesions on axial enhanced MRI. (a, b) At 63 months postoperatively, follow-up MRI revealed two new intraparenchymal lesions across the precentral sulcus: (a) a 3-mm lesion in the middle frontal gyrus (arrow) and (b) a 2-mm lesion in the precentral gyrus (arrow). (c, d) At 65 months (2 months after low-dose SRT), follow-up MRI shows complete resolution of both lesions. MRI; magnetic resonance imaging, SRT; stereotactic radiotherapy

Contrast-enhanced magnetic resonance imaging (MRI) of the spinal cord was performed to assess for further CSF dissemination, but no tumor was detected. These lesions showed minimal shrinkage, consistent with the typical radiobiology of meningiomas (Figures 3c, 3d, 3h). At 63 months postoperatively, contrast-enhanced MRI revealed two new enhancing lesions in the right frontal lobe, extending from the cortical surface into the parenchyma, in areas not previously irradiated (Figures 4a, 4b).

**Figure 5:**
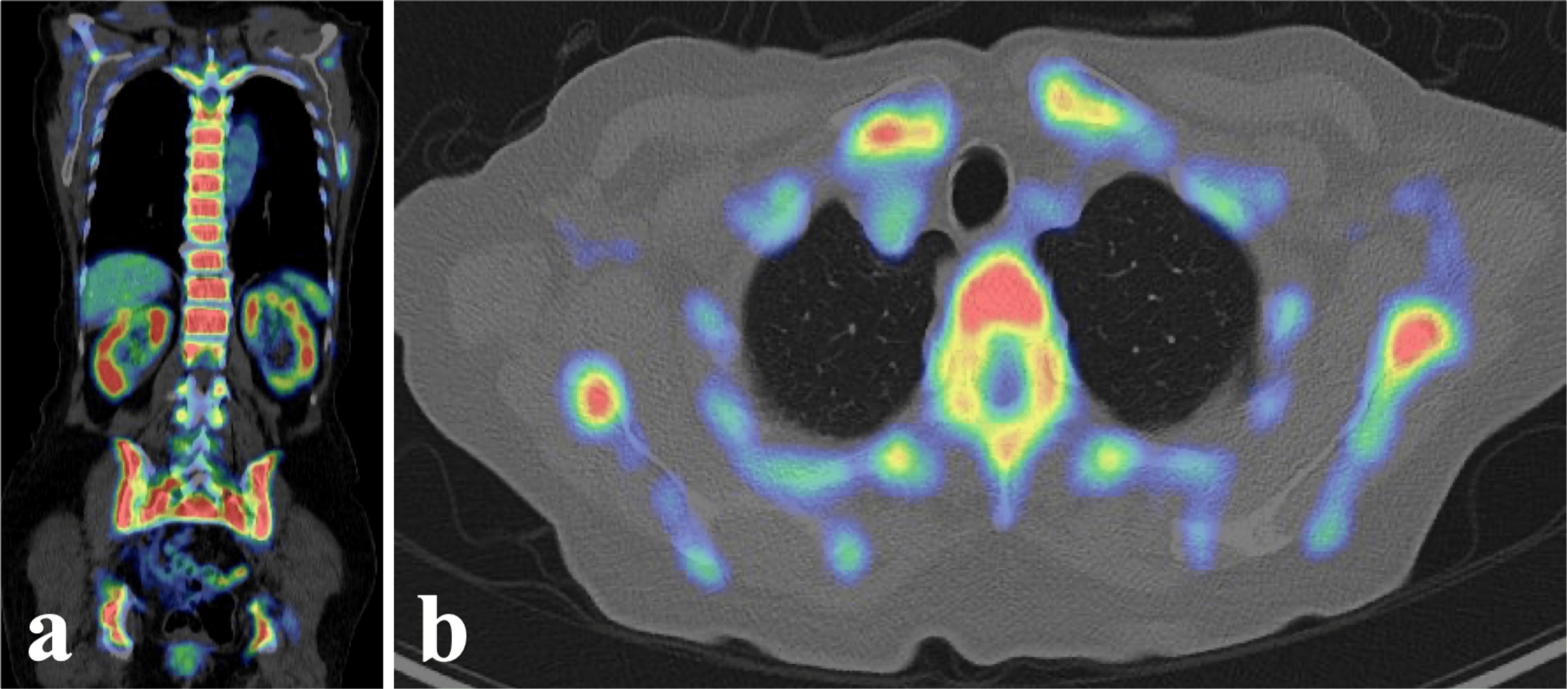
FDG-PET/CT after the fourth SRT (a) Coronal view, (b) Axial view FDG; 2-deoxy-2-(18F) fluoro-D-glucose, PET/CT; positron emission tomography/computed tomography, SRT; stereotactic radiotherapy

A fourth SRT was performed under the presumptive diagnosis of further meningioma dissemination (Figure 2h). Remarkably, follow-up MRI revealed complete resolution of these lesions only two months later (Figures 4c, 4d). This unexpectedly rapid response, highly atypical for meningioma, prompted a systemic investigation for an alternative pathology. Positron emission tomography/computed tomography revealed diffuse 2-deoxy-2-(18F) fluoro-D-glucose uptake in the bone marrow (Figure 5).

No hematological abnormalities were observed until this presentation. The patient was referred to the Department of Hematology and Oncology.

**Figure 6:**
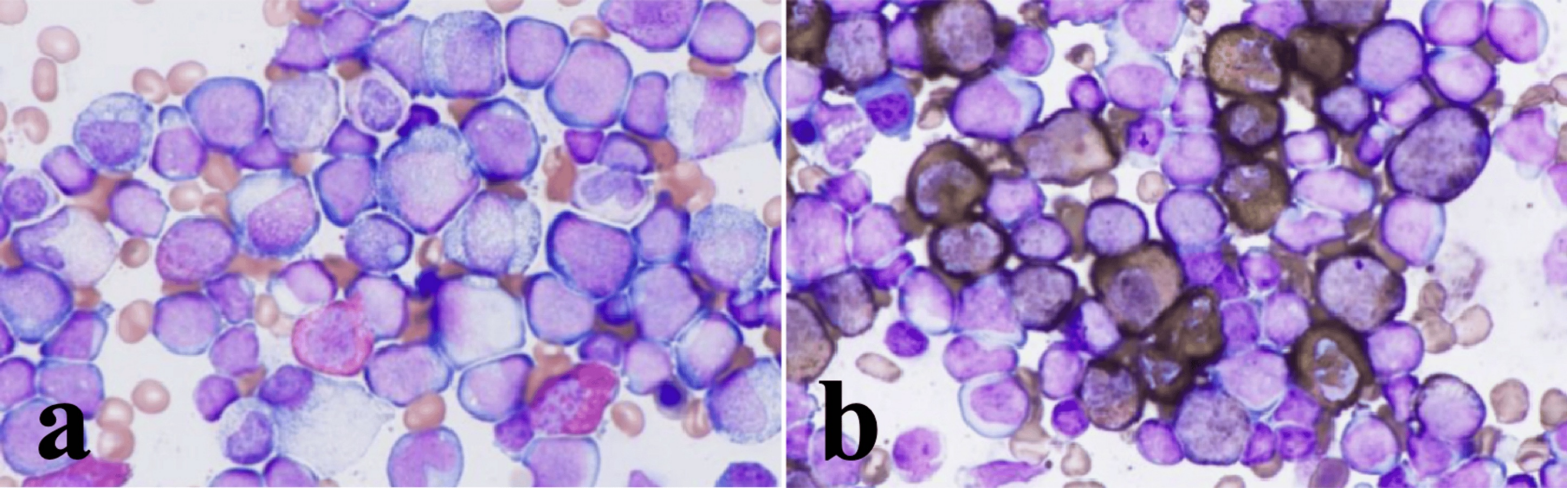
Bone marrow aspirate smear showing leukemic blasts (a) Giemsa stain (x1000): Approximately 56% of the marrow cells are blasts. (b) Myeloperoxidase stain (×1000): Blasts showing positivity in more than 3% of cells.

Although asymptomatic, laboratory findings revealed severe pancytopenia: white blood cell count of 2560/μL (19% blasts), hemoglobin level of 4.2 g/dL, and platelet count of 26000/μL. A bone marrow examination revealed approximately 56% blastoid cells, and normal differentiated cells were suppressed (Figures 6a, 6b).

Immunohistochemical staining revealed co-expression of myeloperoxidase (myeloid lineage) and CD79a/TdT (B-lymphoid lineage), confirming the mixed phenotype diagnosis, with additional markers including CD34 (+), CD117 (+), CD20 (-), CD3 (-), and weak p53 (+). Flow cytometry revealed a positive expression for CD10, CD19, CD22, CD13, CD33, CD34, CD117, cyCD79a, cyTdT, and human leukocyte antigen-DR. A G-banding analysis revealed a 46, XX, t(9;22)(q34.1;q11.2)(20/20). The major bcr-abl mRNA level in peripheral blood was 140000 copies/μgRNA, indicating a high disease burden at diagnosis. Consequently, the patient was diagnosed with Ph+MPAL. Chemotherapy with prednisolone (60 mg/m2) and dasatinib (100 mg/body) was initiated. The therapy was discontinued after 1 month because of a significant pleural effusion as an adverse effect. Post-treatment laboratory examination showed normalization of neutrophil and platelet counts and disappearance of leukemic blasts, meeting criteria for complete remission (CR). After achieving CR, the patient received home-based palliative care. Although the patient experienced several complications, including aspiration pneumonia, cerebral infarction, and hydrocephalus secondary to recurrent meningioma, there was no evidence of Ph+MPAL recurrence until her death 23 months after the cessation of chemotherapy.

## Discussion

IVMs that are not attached to the dura are extremely rare, arising from meningeal tissue within the choroid plexus. CSF dissemination from meningiomas is even rarer, with a reported incidence of 0.9%-4% [5,6]. Most cases occur after surgical resection, as seen in 95.6% of the 45 cases reviewed by Park et al. [5]. Although dissemination is more common in WHO grade II-III tumors, approximately 40% of cases are initially diagnosed as grade I. While we could not histologically confirm malignant transformation in our case, the clinical appearance of dissemination often parallels a transition toward more aggressive behavior. The primary site was often intraventricular, accounting for 23.8% (10/42) [5]. Complete resection of deep intraventricular tumors can be challenging, and piecemeal resection increases the risk of CSF dissemination. In Park et al.'s five cases, the mean time from initial surgery to local recurrence was 2.3 years (range, 2.5 months to 6.9 years), and the mean time to CSF dissemination was 2.9 years (range, 2.5 months to 6.9 years) [5]. Spinal cord dissemination is reported in 78% (35/45) of meningioma CSF dissemination cases, highlighting the need to evaluate the spine when CSF dissemination is suspected [5]. Although multiple surgeries, systemic or intraventricular chemotherapy, and radiotherapy (whole brain, spine, or localized) have been reported for CSF dissemination, no standard treatment strategy has been established [5]. In our case, two superficial lesions located distant from the resection cavity were targeted in the second and third SRT sessions; both grew gradually and showed a typically slow radiation response consistent with the radiobiology of benign meningiomas [7]. However, the two new lesions that appeared 63 months postoperatively exhibited strikingly different behavior. The complete resolution of these lesions within only two months after low-dose SRT was the "diagnostic clue." This "exquisite radiosensitivity" is highly atypical for meningioma and strongly suggests hematologic malignancy, such as intracranial leukemic infiltration, which is characteristically responsive to low-dose radiation due to the high proliferative fraction and inherent radiosensitivity of leukemic blasts.

MPAL accounts for only 1%-5% of all acute leukemias [8], including 2%-5% of adult acute myeloid leukemia and 5%-10% of adult acute lymphoblastic leukemia [9]. To date, no standardized treatment protocol has been established for Ph+MPAL due to its rarity and heterogeneity. Ph+MPAL was previously thought to have an extremely poor prognosis, but in recent years, remission and survival rates have improved dramatically with the combined use of TKIs such as imatinib and dasatinib [10-12]. However, Ph+MPAL in an 82-year-old patient presents significant therapeutic challenges. In our case, a less intensive regimen of dasatinib and prednisolone was selected due to the patient’s age [12]. Although dasatinib was discontinued after one month due to pleural effusion, a known adverse effect of second-generation tyrosine kinase inhibitors [13], the patient achieved and maintained CR for 23 months. This favorable outcome, despite the abbreviated treatment, suggests that the early diagnostic intervention triggered by the atypical imaging response played a crucial role.

A key question is whether this Ph+MPAL represents TRL. TRL develops after treatment for malignant tumors, typically from DNA or chromosomal damage induced by radiation or cytotoxic drugs. TRL caused by radiation is a stochastic effect, occurring without a threshold and manifesting years to decades after exposure [14]. The incidence of TRL is increasing with the growing population of long-term cancer survivors. Five cases of MPAL as TRL have been reported in patients previously treated for seminoma [15], endometrial adenocarcinoma [16], diffuse large B-cell lymphoma [17,18], and neuroblastoma [19]; no cases have been reported after benign tumors. In a review of 26 TRL cases following brain tumor treatment, none occurred after radiotherapy alone [20]. To our knowledge, this is the first reported case of MPAL appearing after localized SRT for a benign meningioma. However, causality cannot be definitively established, and the MPAL may represent a coincidental de novo malignancy rather than a therapy-related event. While the cumulative bone marrow dose from four SRT sessions might have contributed to leukemogenesis, a coincidental development of de novo MPAL cannot be entirely excluded. Regardless of the etiology, this case highlights that clinicians must remain vigilant for secondary or unrelated malignancies during long-term follow-up of brain tumor patients.

In summary, the exquisite radiosensitivity observed in this case served as a critical diagnostic clue. Such a disproportionately rapid response to SRT, which deviates from the known radiobiology of meningioma, should alert clinicians to the possibility of an underlying hematologic malignancy. Clinicians should reconsider the initial diagnosis when a tumor shows an unexpected response to radiotherapy. Early detection of underlying hematologic malignancies, even in elderly patients, can lead to successful remission with tailored, low-intensity treatment regimens.

## Conclusions

We presented a rare case of Ph+MPAL mimicking CSF dissemination of an IVM. Clinicians should reconsider the initial diagnosis when a tumor shows an unexpected response to radiotherapy. Early detection of hematological malignancies is crucial, as even a brief course of targeted therapy may lead to complete remission, even in elderly patients.
